# Embryonic Exposure to Valproic Acid Impairs Social Predispositions of Newly-Hatched Chicks

**DOI:** 10.1038/s41598-018-24202-8

**Published:** 2018-04-12

**Authors:** Paola Sgadò, Orsola Rosa-Salva, Elisabetta Versace, Giorgio Vallortigara

**Affiliations:** 1 Center for Mind/Brain Sciences, University of Trento, Piazza della Manifattura 1, Rovereto, Italy; 2 Department of Biological and Experimental Psychology, School of Biological and Chemical Sciences, Queen Mary University of London, Mile End Road, London, E1 4NS UK

## Abstract

Biological predispositions to attend to visual cues, such as those associated with face-like stimuli or with biological motion, guide social behavior from the first moments of life and have been documented in human neonates, infant monkeys and domestic chicks. Impairments of social predispositions have been recently reported in neonates at high familial risk of Autism Spectrum Disorder (ASD). Using embryonic exposure to valproic acid (VPA), an anticonvulsant associated to increased risk of developing ASD, we modeled ASD behavioral deficits in domestic chicks. We then assessed their spontaneous social predispositions by comparing approach responses to a stimulus containing a face configuration, a stuffed hen, *vs*. a scrambled version of it. We found that this social predisposition was abolished in VPA-treated chicks, whereas experience-dependent mechanisms associated with filial imprinting were not affected. Our results suggest a specific effect of VPA on the development of biologically-predisposed social orienting mechanisms, opening new perspectives to investigate the neurobiological mechanisms involved in early ASD symptoms.

## Introduction

Autism Spectrum Disorder (ASD) comprises a genetically heterogeneous group of neurodevelopmental disabilities characterized by a wide range of impairments in social behaviors. Given their genetic heterogeneity and the complex behavioral traits associated with ASD diagnosis, animal models are essential for the study of the mechanistic bases of these disorders and for development of potential therapies. Despite several studies addressing the importance of early diagnosis and intervention in ASD^1^, to date symptom recognition is achieved after 2 years of age. Delineating the earliest expression of ASD would increase the opportunities for intervention and advance our understanding of the underlying biology; therefore, animal models investigating the early development and mechanistic bases of ASD would have a crucial impact on the development of therapies.

One of the aspects that limit neurodevelopmental studies in existing animal models of ASD is the availability of early social behavioral tests, reliably recapitulating the social impairment shown in the patients. Biological predispositions to attend to social stimuli, without any previous experience (social predispositions thereafter), are the earliest expression of social behavior. Social predispositions have been described in humans^2^, non-human primates^3^ and domestic chicks^4^, as spontaneous, hard-wired, mechanisms that drive visual attention to features associated with social partners^5^. Typical newborn babies show, for instance, preference for faces and face-like configurations^5^ exactly as newly-hatched chicks^6,7^ and naïve infant monkeys do^8^. These inter-species similarities in response to social cues (or more generally to cues of “animacy”) extend to biological motion^9,10^, self-propulsion^11,12^, and speed changes^13^ (though in some cases species differences have been also reported)^14^. Most important, some of the subpallial areas linked to social predispositions have started to be identified^15–17^. Our findings show activation of septal and preoptic area neurons in response to animate motion of a social partner and to speed-changes stimuli^15,18^. The septum could be involved in establishing the emotional valence of these stimuli^19^. In addition, activation of the arcopallium and the nucleus taeniae (partially homolog to some parts of the mammalian amygdala, see Discussion) have been described in response to exposure to the static features of social companions (e.g. a face-like configuration)^17,19^. The human amygdala is involved in processing socially and emotionally salient stimuli and is believed to play a role in early social orienting responses towards face stimuli^20^. Finally, we found differential activation of the intermediate medial mesopallium (IMM), an associative area involved in imprinting learning^21^, in chicks that approached a stuffed hen compared to a control stimulus^16^.

Social predispositions have also been associated with behavioral deficits in ASD. A recent prospective study analyzed these early-emerging mechanisms in neonates (four to ten days old) with a high familial risk of ASD^22^. Measuring visual attention towards face-like stimuli and biological motion cues, impairment in the orienting mechanisms towards these social stimuli was observed in neonates at high-risk for ASD, compared to the typical population. This discovery moves the potential for early ASD assessments to the first moments of life, thus increasing the interest for models reproducing symptoms that can be assessed soon after birth.

Clinical studies have shown that prenatal exposure to the histone deacetylases (HDACs) inhibitor valproic acid (VPA) is associated with neural tube malformations, reduced cognitive function and an increased risk for developing ASD^23^. VPA directly inhibits HDACs^24^, interfering with normal deacetylation of chromatin and causing activation of aberrant gene transcription during development^25^. Given the strong association of VPA treatment with development of social behavioral deficits in humans, animal studies using prenatal exposure to VPA have been conducted, to model the core signs of ASD and to identify the molecular pathways linked to ASD social deficits^26,27^. Despite several investigations devoted to the study of histone acetylation and the effect of HDAC inhibitors on memory and cognition^28,29^, the detrimental effect of these compounds on brain development and social behaviors, and their role in the etiology of ASD is still unclear. Previous studies conducted in chicks showed that VPA can alter aggregative behavior and decrease vocalizations^30^. To investigate the contribution of social predispositions to atypical social behavior related to ASD, in an animal model that allows for controlled experimental conditions, we delivered VPA *in ovo*, in the last week of embryogenesis, and compared the performance of VPA- and vehicle-injected chicks on social predispositions to approach a social stimulus (a stuffed hen), and on affiliative responses mediated by the learning mechanism of filial imprinting.

## Methods

### Ethical statement

All experiments comply with the current Italian and European Community laws for the ethical treatment of animals. The experimental procedures were approved by the Ethical Committee of the University of Trento and licensed by the Italian Health Ministry (permit number 986/2016-PR).

### Chick embryo injections

Freshly fertilized eggs of domestic chicks (*Gallus gallus*), of the Ross 308 (Aviagen) strain, were obtained from a local commercial hatchery (Agricola Berica, Montegalda (VI), Italy), placed in a cold room at 4 °C and maintained in a vertical position for 24–72 h. The eggs were then placed in the dark and incubated at 37.5 °C and 60% relative humidity, with rocking. The first day of incubation was considered embryonic day 0 (E0). Fertilized eggs were then selected by a light test on E14 before injection. Chick embryo injection was performed according to previous reports^30^. Briefly, a small hole was made on the egg shell above the air sac, and 35 μmoles of VPA (Sodium Valproate, Sigma Aldrich) were administered to each fertilized egg, in a volume of 200 μl, by dropping the solution onto the chorioallantoic membrane. Age-matched control eggs were injected using the same procedure with 200 μL of vehicle (double distilled injectable water). After sealing the hole with paper tape, eggs were placed in a rocking incubator (FIEM srl, Italy) until E18, when eggs were placed in a hatching incubator (FIEM srl, Italy). Hatching took place at a temperature of 37.7 °C, with 60% humidity, as previously described^16^. The day of hatching was considered post-hatching day 0 (P0). All subsequent procedures were performed in complete darkness, so that the chicks remained visually inexperienced until the moment of test. Two independent samples of chicks were used for the two experiments described.

### Data availability

All data generated or analyzed during this study are included in this published article and its Supplementary Information files.

## Experiment 1. Social predispositions test

### Rearing conditions, apparatus and stimuli

**Figure 1:**
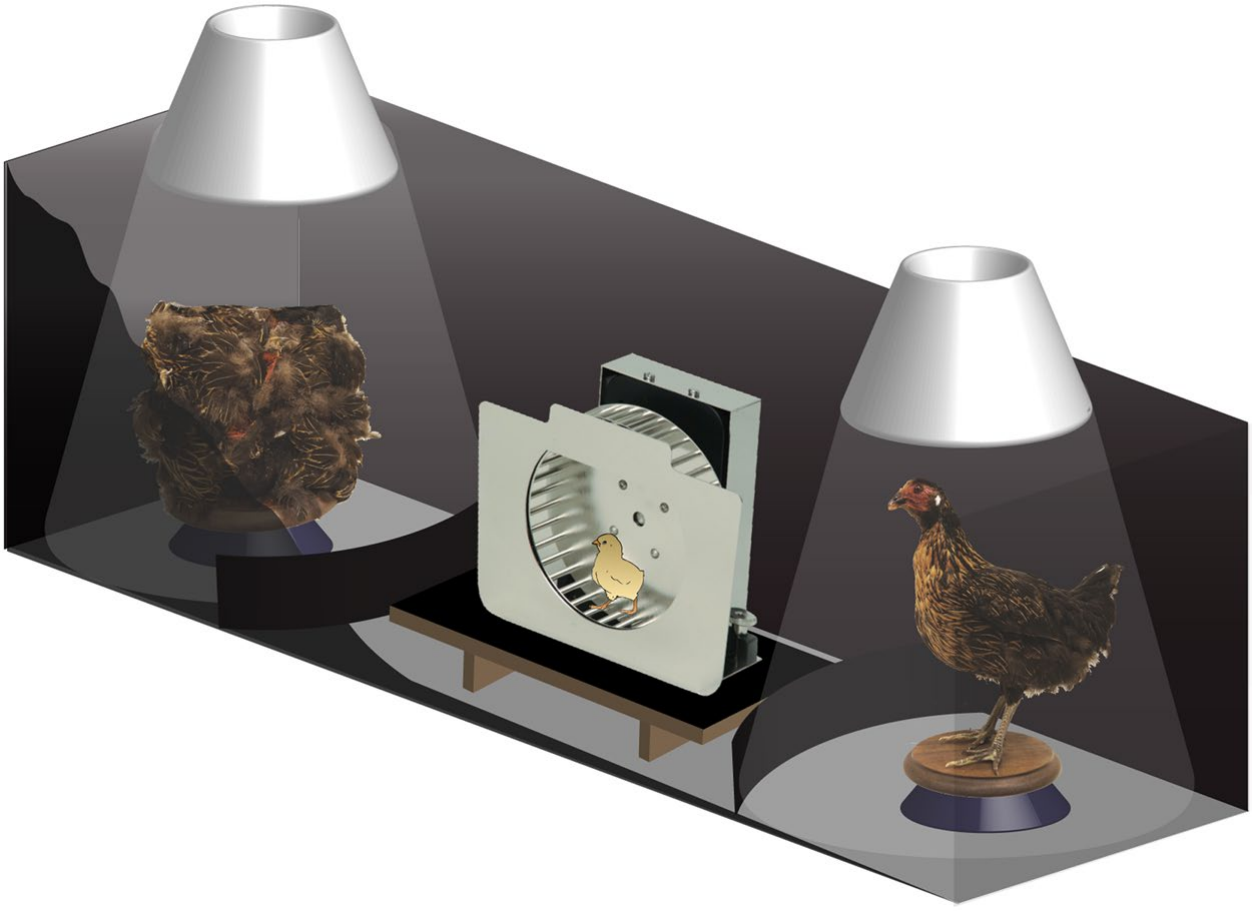
Social predispositions test experimental setup. Schematic illustration of the social predispositions test apparatus. The visually-naïve chick was placed in the running wheel and was free to approach either of the stimuli, both visible at the two ends of the apparatus. The chick’s behavior was video-recorded from above. The stimuli consisted of a stuffed hen and a box, in which features of the hen were dismantled and attached on the sides of the box in a scrambled order^16,32–34^, positioned on two rotating platforms and illuminated from above and by top/front lights (not shown here). Approach to the stimuli was measured by an automated system recording the distance run (number of rotations) in each direction. Running wheel image courtesy of TSE Systems (Germany).

We used the same procedure previously described to assess chicks’ social predispositions^31^. Twenty-four hours after hatching, at post-hatching day 1 (P1), chicks were transferred to individual compartments (11 cm × 11 cm × 25 cm) at the constant temperature of 33 °C. To enhance the expression of the social predispositions, chicks were exposed to acoustic stimulation^31^ inside a dark incubator equipped with a loudspeaker. Non-species-specific sound stimulation was provided using a digitally constructed audio file as previously described^16^. The test apparatus consisted of a running wheel (TSE Systems, Germany) mounted at the center of a 150 cm-long and 46 cm-wide arena, with lateral walls of 45 cm of height^32^. Stimuli were located at the opposite sides of the apparatus, on two rotating platforms (30 rotations per minute), illuminated from above (40 W warm diffused light) and by top/front lights (25 W warm light). The test stimuli consisted in an intact stuffed jungle fowl hen and a box, in which features of the hen were dismantled and attached on the sides of the box in a scrambled order (Fig. 1), as described previously^16,32–34^.

### Test procedure

Chicks’ preferences for a stuffed hen *vs*. a scrambled stuffed hen were tested at P2 for 30 minutes. Each subject was individually extracted from the incubator in complete darkness and carried, in a closed box, to the experimental room. Chicks were then individually placed in the running wheel facing one of the lateral walls, so that they could see both stimuli on the opposite sides of the apparatus. Their approach responses were recorded as distance run (number of wheel rotations) in the direction of each stimulus. Right/left stimulus position, Sex and Treatment groups were balanced between experimental sessions. Each session was video recorded.

## Experiment 2. Filial imprinting test

### Rearing conditions, apparatus and stimuli

Chicks hatched in individual compartments (11 × 8.5 × 14 cm) in complete darkness. Chicks were placed in the imprinting set-up soon after hatching, with water and food *ad libitum*, and exposed to the imprinting stimulus for 3 days. The imprinting set-up consisted of a black box (30 cm × 30 cm × 40 cm) with a monitor (17′′, 60 Hz) mounted on the front wall, displaying one of the imprinting stimuli for 14 hours a day, continuously. During the remaining 10 hours, the screen was black and the lights were off, allowing the normal day-night cycle. Imprinting stimuli consisted of either a blue circle or a red triangle (3.7 cm circle diameter and triangle sides), moving at 1.5 cm/s on a white screen^35,36^. The same two stimuli were used for imprinting test: one (familiar) stimulus that was known to the chicks, and the other one, unfamiliar, that the chicks had never seen before. Chicks were individually tested at P3. The imprinting test apparatus consisted of a 150 cm long, 46 cm wide, 45 cm high arena, equipped with a running wheel (32 cm diameter, 13 cm large, covered with 1 cm of opaque foam on both sides) in the center of the apparatus.

### Test procedure

Chicks’ preferences for the imprinted (familiar) stimulus *vs*. the unfamiliar stimulus were tested at P3 for 20 minutes. Chicks were individually placed in the center of the running wheel, facing one of the lateral walls, so that they could see both stimuli on the opposite sides of the apparatus. The preference for the imprinted and for the unfamiliar stimulus was measured using the distance run (centimeters) towards each stimulus. Right/left stimulus position, Type of imprinting stimulus (red triangle or blue circle), Sex and Treatment groups were balanced between experimental sessions. Each session was video recorded.

### Statistical analysis

To assess social predispositions and imprinting responses independently from motor activity, we calculated for each chick a preference score for the social/imprinted stimulus adjusted for the overall distance run, as

$${\rm{preference}}\,{\rm{score}}=\frac{{\rm{distance}}\,{\rm{towards}}\,{\rm{the}}\,{\rm{social}}\,({\rm{imprinted}})\,{\rm{stimulus}}}{{\rm{overall}}\,{\rm{distance}}\,{\rm{run}}}$$

Values of this ratio range from 1 (full choice for the social/imprinted stimulus) to 0 (full choice for the non-social/unfamiliar stimulus), where 0.5 represents the absence of preference. We assessed differences in motor activity by comparing the overall distance run, regardless of the approached stimulus, for the entire test session. Effect of Treatment, Sex and Type of imprinted stimulus on the preference score was evaluated by multifactorial analysis of variance (ANOVA). For all the tests, significant departures of the preference score from chance level (0.5) were estimated by one-sample two-tailed t-tests. All statistical analyses were performed with IBM SPSS Statistic for Windows (Version 24.0). Alpha was set to 0.05 for all tests.

## Results

**Figure 2:**
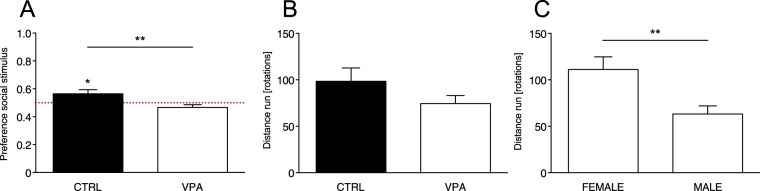
Social predispositions test. Bar graphs of preferences scores and motor activity in the social predispositions test. (**A**) Social preference test for stuffed hen (social stimulus) and non-social stimulus (see Methods for details). Analysis of variance of social preference scores using Treatment and Sex as between-subject factors, revealed a significant main effect of Treatment (line with asterisks), with no other main effects or interactions among the other factors analyzed. Preference scores were significantly different from chance level for the control group, but not for VPA-treated chicks. Asterisks indicate significant departures from chance level, marked by the red line at 0.5. (**B**,**C**) Motor activity in the running wheel. Analysis of variance on number of rotations using Treatment and Sex as between-subject factors, showing (**B**) no significant main effects of Treatment or interaction Treatment × Sex and (**C**) a significant effect of Sex independent of Treatment. Data represent mean ± SEM, *p < 0.05; **p < 0.01.

Previous studies have shown that visually-naïve chicks prefer to approach a social stimulus consisting in a stuffed hen over an artificial object, or even over a scrambled version of the same stimulus^32^. We performed the same test in visually-naïve VPA- and vehicle-injected domestic chicks and assessed their predisposed preference to approach a naturalistic stimulus consisting of a stuffed hen over a “non-social” stimulus, in which features of the hen were dismantled and attached on the sides of a box in scrambled order^32^ (see Methods for details, see Fig. 1). To evaluate social predispositions, we calculated the preference score for the social stimulus as the proportion of running wheel rotations toward the stuffed hen (see Methods for details). Results of the analysis of variance showed an effect of Treatment on the chicks’ preference for the social stimulus (F_1,58_ = 7.708, p = 0.007), and no significant effect of Sex (F_1,58_ = 0.022, p = 0.883) or their interaction (Treatment × Sex (F_1,58_ = 0.050, p = 0.823). In the control group, the preference for approaching the stuffed hen was similar to what previously observed^16,34^ and significantly different from chance (t_28_ = 2.191, p = 0.037, preference score *M* = 0.564, *SEM* = 0.158, *N* = 29, 16 males and 13 females; Fig. 2A). On the contrary, VPA exposure significantly reduced the preference for the stuffed hen compared to controls and the preference was not significantly different from chance (t_32_ = −1.684, p = 0.102, preference score *M* = 0.466, *SEM* = 0.116, *N* = 33, 18 males and 15 females, Fig. 2A). In line with this observation, the average preference score for the social stimulus was significantly different from chance level only for the control group and not for VPA-treated chicks (Fig. 2A). Thus, VPA exposure significantly reduces the predisposed preference for the stuffed hen to chance level. On the contrary, in motor activity, measured as the overall number of rotations in the running wheel, we did not observe a significant effect of Treatment (F_1,58_ = 1.385, p = 0.244) or interaction Treatment × Sex (F_1,58_ = 0.021, p = 0.884) but a significant effect of Sex (F_1,58_ = 9.707, p = 0.003; Fig. 2B). Females were significantly more active than males, irrespective of Treatment (overall number of rotations for females *M* = 111.14, *SEM* = 71.94, *N* = 28 and for males *M* = 63.35, *SEM* = 48.14, *N* = 34; Fig. 2C).

**Figure 3:**
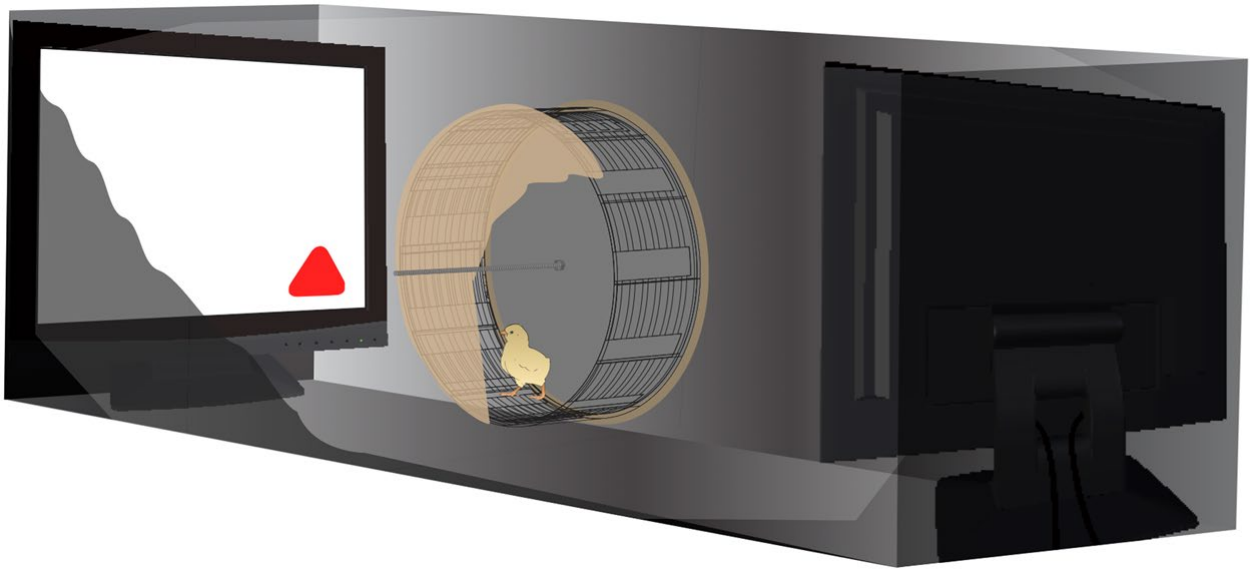
Filial imprinting test experimental setup. Schematic illustration of the filial imprinting test apparatus. After imprinting, each chick was placed in the running wheel and tested for its approach response to the imprinted stimulus (red triangle shown) or the unfamiliar stimulus, never seen before. Monitors used to present the stimuli were located at the far ends of the apparatus. Approach to the stimuli was measured by an automated system recording the distance run (centimeters) in each direction.

**Figure 4:**
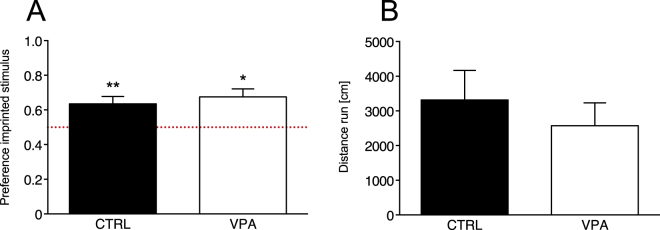
Filial imprinting test. Bar graphs of preferences scores and motor activity in the filial imprinting test. (**A**) Analysis of variance using Treatment and Sex as between-subject factors, revealed no significant main effect or interactions. The mean preference scores for the imprinted stimulus were significantly different from chance level for both treatment groups. Asterisks indicate significant departures from chance level, which is marked by the red line at 0.5. In both treatment groups preference scores >0.5, indicating preference for the imprinted stimulus. (**B**) Analysis of variance on motor activity during the test did not detect any significant main effect of Treatment, Sex or their interaction in the motor activity of the chicks during the imprinting tests in the running wheel. Data represent mean ± SEM, *p < 0.05; **p < 0.01.

To investigate whether VPA specifically affected social predispositions or impaired affiliative responses and cognitive abilities in general, we tested the effect of VPA also on filial imprinting. Differently from social predispositions, the learning mechanism of filial imprinting orients affiliative responses of chicks after previous exposure to a conspicuous stimulus^37,38^. Although it has been suggested that, in the wild, social predispositions might guide the learning process of filial imprinting by orienting the initial responses of chicks towards the mother hen, imprinting has a different neurobiological basis than social predispositions^39,40^. We exposed VPA- and vehicle-injected chicks to filial imprinting with artificial 2D objects (see Methods, Fig. 3), and subsequently measured their learned preference for the imprinting stimulus *vs*. an unfamiliar stimulus. The chicks’ approach to the imprinting stimulus was not significantly different between treatment groups and sexes, nor their interaction, as shown by the analysis of variance (Treatment (F_1,47_ = 0.037, p = 0.849), Sex (F_1,47_ = 0.009, p = 0.924), Treatment × Sex (F_1,47_ = 0.331, p = 0.568)). The mean preference scores for the imprinted stimulus were significantly different from chance level for both treatment groups (CTRL: t_25_ = 3.173, p = 0.004; VPA: t_28_ = 3.743, p = 0.001; preference score for controls *M* = 0.635, *SEM* = 0.042, *N* = 26, 12 males and 14 females and for VPA-treated chicks *M* = 0.675, *SEM* = 0.046, *N* = 29, 16 males and 13 females; Fig. 4A), indicating no significant effect of VPA on the learning mechanisms of imprinting. Both groups successfully imprinted on either of the stimuli, and no difference was detected in motor activity in the analysis of variance (Treatment (F_1,47_ = 0.666, p = 0.418), Sex (F_1,47_ = 0.421, p = 0.520), Treatment × Sex (F_1,47_ < 0.0001, p = 0.992); Fig. 4B), suggesting that VPA exposure did not cause major impairments in fundamental motivational, perceptual, motor and cognitive functions.

## Discussion

Several studies have shown that early intervention can reduce the incidence and severity of symptoms in ASD children as young as 12 months of age^41–43^. Early diagnosis and timely interventions for ASD patients could therefore have a crucial impact on the development of therapies. Establishing animal models to investigate the early development and mechanistic bases of ASD could not only improve therapeutic interventions but also shed light on the biological mechanisms underlying ASD. Precocial social species^44^ that exhibit early social predispositions at birth, such as domestic chicks, are a more convenient model to investigate these early-emerging social behaviors, compared to rodents. The domestic chick model enables the expploration of the visual social predispositions observed also in human neonates^2,6,7,9–12,22^, the assessment of the genetic bases of early predispositions^33^, the investigation of the neurobiological bases of social predispositions^16–18^ and an accurate control of the environment in which embryos and chicks develop^45^. In spite of this, to our knowledge, no work has been previously carried out on the possibility that VPA, a compound used to induce ASD-like behavioral deficits in many vertebrate species^46–50^ including domestic chicks^30^, may affect early social predispositions. Combining this novel class of inter-species behavioral mechanisms with the evolutionary conserved effect of VPA on social behaviors, we present a new tool, of potential translational value, to investigate the mechanistic bases of ASD social deficits with the aid of early behavioral markers analogous to those used in human studies.

Our results show a detrimental effect of VPA on the well documented predisposition to approach a social stimulus (stuffed hen) over a comparable “non-social” stimulus (scrambled hen-like box)^32,34^. On the contrary, when we investigated affiliative responses mediated by the experience-dependent mechanism of filial imprinting, we found that VPA did not impair these responses. These results indicate that, despite the fact that VPA disrupted the chicks’ predisposition to preferentially approach the social stimulus, the brain capacity to activate basic experience-dependent learning mechanisms remains intact.

These data suggest a specific effect of VPA on social behaviors^30^ and in particular, on social predispositions. Indeed, despite its wide biological targets, acute exposure to VPA during late embryogenesis has been shown to act preferentially on social behaviors, and to spare general cognitive and motor functions in many vertebrate species^51^. However, while the results of filial imprinting indicate that VPA-treated chicks display adequate basic motivational, perceptual, visuomotor and cognitive functions, we cannot rule out subtler perceptual or cognitive deficits that might interfere with social predispositions. For example, the discrimination of the stimuli used in the social predispositions experiment, may require finer perceptual processing, compared to the discrimination of the artificial stimuli used in the imprinting experiment. Nonetheless, in both experiments, motor activity did not differ between VPA- and vehicle-injected chicks even though motor responses of VPA-treated chicks were significantly less oriented towards the social stimulus in the social predisposition test. Additional studies should investigate the various aspects of VPA-treated chicks’ visual and cognitive functions. Experiments in this direction could investigate the differential imprinting ability of controls and VPA-treated chicks to stimuli that are favoured by social predispositions, compared to stimuli that are not (e.g., the stuffed hen compared to the scrambled hen-like box). Further studies should also investigate the specific mechanisms and brain areas/circuits impaired by VPA exposure. It should be noted that the mechanisms through which social predispositions normally act have not been identified yet. Thus, whether perceptual-attentional mechanisms that modulate the saliency of different features of the stimuli play a role, or motivational and valence-attribution mechanisms are involved, is not yet clear. However, given that individuals with ASD tend to have a more locally-oriented processing style, rather than paying attention to the global appearance of visual configurations^52^, it cannot be excluded that VPA exposure could modify the chicks’ processing style in the same direction.

Previous studies have shown that domestic chicks’ preference responses, such as those analyzed in our study, are elicited by the head and neck region of the stuffed hen^32^, in a striking analogy to faces and the face-like patterns that are known to elicit a strong preference in human neonates^4,6^, and are altered in newborn at high risk of ASD^22^. The role of poor orienting and attention to such important social stimuli during early infancy have been suggested in several instances^53–55^. Social predispositions represent at present the earliest emerging social behaviors in humans. Impairments in these domains could result in limited interests for relevant social stimuli at the time of birth^56^, compromising the typical developmental trajectories of the social brain and contributing to the appearance of ASD symptoms^4^. In line with this hypothesis, recent data have shown impairments in these orienting mechanisms in human neonates at high-risk for ASD^22^. Our data further support these results in an animal model, showing the suppression of a predisposed social preference in domestic chicks exposed to VPA. Furthermore, our data may indicate that VPA exposure in domestic chicks affects development of the social brain circuits involved in social predispositions. In fact, recent studies have shown a response of some nodes of the “social behavior” and “social decision-making” networks during the very first exposure of visually naïve chicks to conspecifics, or to some elementary visual properties that elicit their spontaneous social preferences^17,18^. These data suggest that development and tuning of those networks crucial for the control of adult social behavior might be shaped by early visual inputs that the organism receives from social companions, thanks to its social predispositions and to the species-typical structure of the environment. Previous reports have found differential activation of the intermediate medial mesopallium (IMM, an area involved in filial imprinting learning)^21^ in chicks that approached a stuffed hen compared to a control stimulus^16^. Moreover, recent findings have shown activation of septal and preoptic area neurons in response to animate motion of a social partner and to speed-changes stimuli^15^. The septum could be involved in establishing the emotional valence of these stimuli^19^. In addition, activation of the arcopallium and the nucleus taeniae have been shown in response to exposure to the static features of social companions (e.g. a face-like configuration)^17^, as indicated by the fact that the same differential activation could not be detected in control chicks exposed to an inanimate stimulus presenting an equivalent face-like configuration (a taxidermised dummy chick)^17^. In humans, the amygdala is involved in early social orienting responses towards face stimuli^20^. In birds, at least part of the arcopallium and the nucleus taeniae are considered homologs of parts of the mammalian amygdala complex^57^. However, the precise extent and nature of these homologies are still highly debated^58–61^. While the correspondence of the avian nucleus taeniae, the posterior arcopallium and the subpallial amygdala with parts of the mammalian extended or medial subpallial amygdala are relatively well accepted^62^, a clear picture for the rest of the arcopallium is still missing^63,64^. For instance, other parts of the arcopallium are considered to be premotor in nature, although it has been recently shown that they also process social information^65^ and respond to visual stimuli^66^. Interestingly, while the arcopallium receives projections from the tectofugal visual pathway and has visually responsive neurons, there is no evidence that the septum or the preoptic areas are directly involved in visual processing^66,67^. Visually modulated information could arrive to the septum indirectly from the visual Wulst, through the dorsal hippocampus^68^. Also, inputs from other subtelencephalic regions implicated in unlearned stimulus recognition, such as the pretectal nucleus, could modulate septal activation^69^. Preoptic area’s activation could also be mediated by interconnections with other areas of the social behavior network, to which it belongs.

Altogether, we believe that the model we propose in this study may represent a new tool of potential translational value to investigate the mechanistic bases of social predispositions and their role in early ASD symptoms, and to develop early therapeutic interventions for social behavioral deficits. Further studies should clarify whether VPA effect extends to other social predispositions, such as preferences for dynamic stimuli that change in speed^13,34^, whether this substance has a disruptive or a delaying effect on social predispositions^70^, while future research will explore the possibilities to rescue social responses after exposure to VPA.

## Electronic supplementary material

Dataset1

## References

[1] Sacrey LAR, Bennett JA, Zwaigenbaum L (2015). Early Infant Development and Intervention for Autism Spectrum Disorder. J Child Neurol.

[2] Morton J, Johnson MH (1991). CONSPEC and CONLERN: a two-process theory of infant face recognition. Psychol Rev.

[3] Sugita Y (2009). Innate face processing. Curr Opin Neurobiol.

[4] Di Giorgio, E. *et al*. Filial responses as predisposed and learned preferences: Early attachment in chicks and babies. *Behav Brain Res* **325**, 90–104 (2017). 10.1016/j.bbr.2016.09.018 27616345

[5] Johnson MH, Dziurawiec S, Ellis H, Morton J (1991). Newborns’ preferential tracking of face-like stimuli and its subsequent decline. Cognition.

[6] Rosa Salva O, Regolin L, Vallortigara G (2010). Faces are special for newly hatched chicks: evidence for inborn domain-specific mechanisms underlying spontaneous preferences for face-like stimuli. Dev Sci.

[7] Rosa Salva O, Farroni T, Regolin L, Vallortigara G, Johnson MH (2011). The Evolution of Social Orienting: Evidence from Chicks (Gallus gallus) and Human Newborns. PLoS ONE.

[8] Sugita Y (2008). Face perception in monkeys reared with no exposure to faces. Proc Natl Acad Sci USA.

[9] Simion F, Regolin L, Bulf H (2008). A predisposition for biological motion in the newborn baby. Proc Natl Acad Sci USA.

[10] Vallortigara G, Regolin L, Marconato F (2005). Visually Inexperienced Chicks Exhibit Spontaneous Preference for Biological Motion Patterns. PLoS Biol.

[11] Mascalzoni E, Regolin L, Vallortigara G (2010). Innate sensitivity for self-propelled causal agency in newly hatched chicks. Proc Natl Acad Sci USA.

[12] Di Giorgio E, Lunghi M, Simion F, Vallortigara G (2017). Visual cues of motion that trigger animacy perception at birth: the case of self-propulsion. Dev Sci.

[13] Rosa Salva O, Grassi M, Lorenzi E, Regolin L, Vallortigara G (2016). Spontaneous preference for visual cues of animacy in naïve domestic chicks: The case of speed changes. Cognition.

[14] Versace E, Schill J, Nencini AM, Vallortigara G (2016). Naïve Chicks Prefer Hollow Objects. PLoS ONE.

[15] Lorenzi E, Mayer U, Rosa Salva O, Vallortigara G (2017). Dynamic features of animate motion activate septal and preoptic areas in visually naïve chicks (Gallus gallus). Neuroscience.

[16] Mayer U, Rosa Salva O, Lorenzi E, Vallortigara G (2016). Social predisposition dependent neuronal activity in the intermediate medial mesopallium of domestic chicks (Gallus gallus domesticus). Behav Brain Res.

[17] Mayer U, Rosa Salva O, Vallortigara G (2017). First exposure to an alive conspecific activates septal and amygdaloid nuclei in visually-naïve domestic chicks (Gallus gallus). Behav Brain Res.

[18] Mayer U, Rosa Salva O, Morbioli F, Vallortigara G (2017). The motion of a living conspecific activates septal and preoptic areas in naive domestic chicks (Gallus gallus). Eur J Neurosci.

[19] O’Connell LA, Hofmann HA (2011). The vertebrate mesolimbic reward system and social behavior network: a comparative synthesis. J Comp Neurol.

[20] Johnson MH (2005). Subcortical face processing. Nat Rev Neurosci.

[21] Horn G (2004). Pathways of the past: the imprint of memory. Nat Rev Neurosci.

[22] Di Giorgio E (2016). Difference in Visual Social Predispositions Between Newborns at Low- and High-risk for Autism. Sci. Rep..

[23] Christensen J (2013). Prenatal valproate exposure and risk of autism spectrum disorders and childhood autism. JAMA.

[24] Phiel CJ (2001). Histone deacetylase is a direct target of valproic acid, a potent anticonvulsant, mood stabilizer, and teratogen. J Biol Chem.

[25] Jergil M, Kultima K, Gustafson A-L, Dencker L, Stigson M (2009). Valproic acid-induced deregulation *in vitro* of genes associated *in vivo* with neural tube defects. Toxicol. Sci..

[26] Nagode DA (2017). Abnormal Development of the Earliest Cortical Circuits in a Mouse Model of Autism Spectrum Disorder. Cell Rep.

[27] Choi CS (2016). The transgenerational inheritance of autism-like phenotypes in mice exposed to valproic acid during pregnancy. Sci. Rep..

[28] Morris MJ, Karra AS, Monteggia LM (2010). Histone deacetylases govern cellular mechanisms underlying behavioral and synaptic plasticity in the developing and adult brain. Behavioural Pharmacology.

[29] McGowan PO, Roth TL (2015). Epigenetic pathways through which experiences become linked with biology. Dev Psychopathol.

[30] Nishigori H (2013). Impaired social behavior in chicks exposed to sodium valproate during the last week of embryogenesis. Psychopharmacology (Berl.).

[31] Egorova OV, Anokhin KV (2003). Experimental Analysis of the Processes of Systems Genesis: Expression of the c-fos Gene in the Chick Brain during Treatments Inducing the Development of the Species-Specific Results-of-Action Acceptor. Neurosci Behav Physiol.

[32] Johnson MH, Horn G (1988). Development of filial preferences in dark-reared chicks. Animal Behaviour.

[33] Versace E, Fracasso I, Baldan G, Dalle Zotte A, Vallortigara G (2017). Newborn chicks show inherited variability in early social predispositions for hen-like stimuli. Sci. Rep..

[34] Rosa Salva O, Mayer U, Vallortigara G (2015). Roots of a social brain: developmental models of emerging animacy-detection mechanisms. Neurosci Biobehav Rev.

[35] Nakamori T, Maekawa F, Sato K, Tanaka K, Ohki-Hamazaki H (2013). Neural basis of imprinting behavior in chicks. Dev Growth Differ.

[36] Versace E (2017). Spontaneous generalization of abstract multimodal patterns in young domestic chicks. Anim Cogn.

[37] Bateson P (2015). Thirty years of collaboration with Gabriel Horn. Neurosci Biobehav Rev.

[38] Solomonia RO, McCabe BJ (2015). Molecular mechanisms of memory in imprinting. Neuroscience & Biobehavioral Reviews.

[39] Horn G, McCabe BJ (1984). Predispositions and preferences. Effects on imprinting of lesions to the chick brain. Animal Behaviour.

[40] McCabe BJ, Horn G, Bateson PP (1981). Effects of restricted lesions of the chick forebrain on the acquisition of filial preferences during imprinting. Brain Res.

[41] Webb SJ, Jones EJH, Kelly J, Dawson G (2014). The motivation for very early intervention for infants at high risk for autism spectrum disorders. Int J Speech Lang Pathol.

[42] Dawson G (2012). Early behavioral intervention is associated with normalized brain activity in young children with autism. J Am Acad Child Adolesc Psychiatry.

[43] Rogers SJ (2012). Effects of a brief Early Start Denver model (ESDM)-based parent intervention on toddlers at risk for autism spectrum disorders: a randomized controlled trial. J Am Acad Child Adolesc Psychiatry.

[44] Versace, E. Precocial. In *Encyclopedia of Animal Cognition and Behavior* **66**, 1–3 Springer International Publishing, (2017).

[45] Versace E, Vallortigara G (2015). Origins of Knowledge: Insights from Precocial Species. Front Behav Neurosci.

[46] Nicolini, C. & Fahnestock, M. The valproic acid-induced rodent model of autism. *Exp Neurol* **299**, 217–27 (2018). 10.1016/j.expneurol.2017.04.017 28472621

[47] Roullet FI, Lai JKY, Foster JA (2013). In utero exposure to valproic acid and autism — A current review of clinical and animal studies. Neurotoxicology and Teratology.

[48] Liu X (2016). Social Preference Deficits in Juvenile Zebrafish Induced by Early Chronic Exposure to Sodium Valproate. Front Behav Neurosci.

[49] Zimmermann FF, Gaspary KV, Leite CE, De Paula Cognato G, Bonan CD (2015). Embryological exposure to valproic acid induces social interaction deficits in zebrafish (Danio rerio): A developmental behavior analysis. Neurotoxicology and Teratology.

[50] Markram H (2007). The intense world syndrome – an alternative hypothesis for autism. Front Neurosci.

[51] Schneider T, Przewłocki R (2005). Behavioral alterations in rats prenatally exposed to valproic acid: animal model of autism. Neuropsychopharmacology.

[52] Happé F, Frith U (2006). The weak coherence account: detail-focused cognitive style in autism spectrum disorders. J Autism Dev Disord.

[53] Johnson MH, Senju A, Tomalski P (2015). The two-process theory of face processing: modifications based on two decades of data from infants and adults. Neurosci Biobehav Rev.

[54] Dawson G, Webb SJ, McPartland J (2005). Understanding the nature of face processing impairment in autism: insights from behavioral and electrophysiological studies. Dev Neuropsychol.

[55] Senju A, Johnson MH (2009). Atypical eye contact in autism: models, mechanisms and development. Neurosci Biobehav Rev.

[56] Webb, S. J., Neuhaus, E. & Faja, S. Face perception and learning in autism spectrum disorders. *Q J Exp Psychol* **70**, 970–86 (2017). 10.1080/17470218.2016.1151059 PMC5026554 26886246

[57] Reiner A (2004). Revised Nomenclature for Avian Telencephalon and Some Related Brainstem Nuclei. J Comp Neurol.

[58] Bruce LL, Neary TJ (1995). The Limbic System of Tetrapods - a Comparative-Analysis of Cortical and Amygdalar Populations. BBE.

[59] Puelles L (2000). Pallial and subpallial derivatives in the embryonic chick and mouse telencephalon, traced by the expression of the genes Dlx-2, Emx-1, Nkx-2.1, Pax-6, and Tbr-1. J Comp Neurol.

[60] Jarvis ED (2005). Opinion: Avian brains and a new understanding of vertebrate brain evolution. Nat Rev Neurosci.

[61] Butler AB, Reiner A, Karten HJ (2011). In Annals of the New York Academy of Sciences.

[62] Yamamoto K, Sun Z, Wang HB, Reiner A (2005). Subpallial amygdala and nucleus taeniae in birds resemble extended amygdala and medial amygdala in mammals in their expression of markers of regional identity. Brain Res Bull.

[63] Hanics J, Teleki G, Alpár A, Székely AD, Csillag A (2016). Multiple amygdaloid divisions of arcopallium send convergent projections to the nucleus accumbens and neighboring subpallial amygdala regions in the domestic chicken: a selective pathway tracing and reconstruction study. Brain Struct Funct.

[64] Herold C, Paulitschek C, Palomero-Gallagher N, Güntürkün O, Zilles K (2018). Transmitter receptors reveal segregation of the arcopallium/amygdala complex in pigeons (Columba livia). J Comp Neurol.

[65] Xin Q, Ogura Y, Uno L, Matsushima T (2017). Selective contribution of the telencephalic arcopallium to the social facilitation of foraging efforts in the domestic chick. Eur J Neurosci.

[66.] Scarf D, Stuart M, Johnston M, Colombo M (2016). Visual response properties of neurons in four areas of the avian pallium. J. Comp. Physiol. A Neuroethol. Sens. Neural. Behav. Physiol..

[67] Benowitz LI, Karten HJ (1976). Organization of the tectofugal visual pathway in the pigeon: a retrograde transport study. J Comp Neurol.

[68] Atoji W, Wild JM, Yamamoto Y, Suzuki Y (2002). Intratelencephalic connections of the hippocampus in pigeons (Columba livia). J Comp Neurol.

[69] Montagnese CM, Zachar G, Bálint E, Csillag A (2008). Afferent connections of septal nuclei of the domestic chick (Gallus domesticus): a retrograde pathway tracing study. J Comp Neurol.

[70] Lennartsson A (2015). Remodeling of retrotransposon elements during epigenetic induction of adult visual cortical plasticity by HDAC inhibitors. Epigenetics & Chromatin.

